# Mental Disorders

**Published:** 1836-01

**Authors:** 


### MENTAL DISORDERS.

### MORAL MANAGEMENT OF THE INSANE.

[The following striking account of a scene in the Bedlam of Paris is extracted from a paper read at the Academy of Sciences by the son of the celebrated Pinel, describing an act of his father's, which deserves everlasting honour, from the wisdom, courage, and humanity which it displays.]

Towards the end of 1792, Pinel, after having many times urged the government to allow him to unchain the maniacs of the Bicêtre, but in vain, went himself to the authorities, and with much earnestness and warmth advocated the removal of this monstrous abuse. Couthon, a member of the commune, gave way to M. Pinel's arguments, and agreed to meet him at the Bicêtre. Couthon then interrogated those who were chained, but the abuse he received, and the confused sounds of cries, vociferations, and clanking of chains in the filthy and damp cells, made him recoil from Pinel's proposition. "You may do what you will with them," said he, "but I fear you will become their victim." Pinel instantly commenced his undertaking. There were about fifty whom he considered might without danger to the others be unchained, and he began by releasing twelve, with the sole precaution of having previously prepared the same number of strong waistcoats, with long sleeves, which could be tied behind the back, if necessary. The first man on whom the experiment was to be tried was an English captain, whose history no one knew, as he had been in chains forty years. He was thought to be one of the most furious among them; his keepers approached him with caution, as he had in a fit of fury killed one of them on the spot with a blow from his manacles. He was chained more rigorously than any of the others. Pinel entered his cell unattended, and calmly said to him, "Captain, I will order your chains to be taken off, and give you liberty to walk in the court, if you will promise me to behave well and injure no one." "Yes, I promise you," said the maniac; "but you are laughing at me, you are all too much afraid of me." "I have six men," answered Pinel, "ready to enforce my commands, if necessary. Believe me then on my word, I will give you your liberty if you will put on this waistcoat"

He submitted to this willingly, without a word: his chains were removed, and the keepers retired, leaving the door of his cell open. He raised himself many times from his seat, but fell again on it, for he had been in a sitting posture so long that he had lost the use of his legs; in a quarter of an hour he succeeded in maintaining his balance, and with tottering steps came to the door of his dark cell. His first look was at the sky, and he cried out enthusiastically, "How beautiful!" During the rest of the day he was constantly in motion, walking up and down the staircases, and uttering short exclamations of delight. In the evening he returned of his own accord into his cell, where a better bed than he had been accustomed to had been prepared for him, and he slept tranquilly. During the two succeeding years which he spent in the Bicêtre, he had no return of his previous paroxysms, but even rendered himself useful by exercising a kind of authority over the insane patients, whom he ruled in his own fashion.

The next unfortunate being whom Pinel visited was a soldier of the French guards, whose only fault was drunkenness: when once he lost self-command by drink he became quarrelsome and violent, and the more dangerous from his great bodily strength. From his frequent excesses, he had been discharged from his corps, and he had speedily dissipated his scanty means. Disgrace and misery so depressed him that he became insane: in his paroxysms he believed himself a general, and fought those who would not acknowledge his rank. After a furious struggle of this sort, he was brought to the Bicêtre in a state of the greatest excitement. He had now been chained for ten years, and with greater care than the others, from his having frequently broken his chains with his hands only. Once when he broke loose, he defied all his keepers to enter his cell until they had each passed under his legs; and he compelled eight men to obey this strange command. Pinel, in his previous visits to him, regarded him as a man of original good nature, but under excitement, incessantly kept up by cruel treatment; and he had promised speedily to ameliorate his condition, which promise alone had made him more calm. Now he announced to him that he should be chained no longer, "and, to prove that he had confidence in him, and believed him to be a man capable of better things, he called upon him to assist in releasing those others who had not reason like himself; and promised, if he conducted himself well, to take him into his own service." The change was sudden and complete. No sooner was he liberated than he became obliging and attentive, following with his eye every motion of Pinel, and executing his orders with as much address as promptness: he spoke kindly and reasonably to the other patients; and during the rest of his life was entirely devoted to his deliverer. And "I can never hear without emotion (says Pinel's son) the name of this man, who some years after this occurrence shared with me the games of my childhood, and to whom I shall feel always attached."

In the next cell were three Prussian soldiers, who had been in chains for many years, but on what account no one knew. They were in general calm and inoffensive, becoming animated only when conversing together in their own language, which was unintelligible to others. They were allowed the only consolation of which they appeared sensible,—to live together. The preparations taken to release them alarmed them, as they imagined the keepers were come to inflict new severities; and they opposed them violently when removing their irons. When released they were not willing to leave their prison, and remained in their habitual posture. Either grief or loss of intellect had rendered them indifferent to liberty.

Near them was an old priest, who was possessed with the idea that he was Christ: his appearance indicated the vanity of his belief; he was grave and solemn; his smile soft and at the same time severe, repelling all familiarity; his hair was long and hung on each side of his face, which was pale, intelligent, and resigned. On his being once taunted with a question that "if he was Christ he could break his chains," he solemnly replied, "Frustra tentaris Dominum tuum." His whole life was a romance of religious excitement. He undertook on foot pilgrimages to Cologne and Rome; and made a voyage to America for the purpose of converting the Indians: his dominant idea became changed into actual mania, and on his return to France he announced himself as the Saviour. He was taken by the police before the archbishop of Paris, by whose orders he was confined in the Bicêtre as either impious or insane. His hands and feet were loaded with heavy chains, and during twelve years he bore with exemplary patience this martyrdom and constant sarcasms. Pinel did not attempt to reason with him, but ordered him to be unchained in silence, directing at the same time that every one should imitate the old man's reserve, and never speak to him. This order was rigorously observed, and produced on the patient a more decided effect than either chains or a dungeon: he became humiliated by this unusual isolation, and after hesitating for a long time, gradually introduced himself to the society of the other patients. From this time his notions became more just and sensible, and in less than a year he acknowledged the absurdity of his previous prepossession, and was dismissed from the Bicêtre.

In the course of a few days, Pinel released 53 maniacs from their chains: among them were men of all conditions and countries; workmen, merchants, soldiers, lawyers, &c. The result was beyond his hopes. Tranquillity and harmony succeeded to tumult and disorder; and the whole discipline was marked with a regularity and kindness which had the most favorable effect on the insane themselves; rendering even the most furious more tractable.

*Memoires de l'Academie de Médecine*, t. v. f. 1, 1835.

